# Optimization of arc quenching parameters for enhancing surface hardness and line width in S45C steel using Taguchi method

**DOI:** 10.1371/journal.pone.0314648

**Published:** 2024-12-02

**Authors:** Van-Thuc Nguyen, Pham Son Minh, Hung-Son Dang, Nguyen Ho

**Affiliations:** 1 HCMC University of Technology and Education, Ho Chi Minh City, Vietnam; 2 Faculty of Mechanical Engineering, HCMC University of Technology and Education, Ho Chi Minh City, Vietnam; Himachal Pradesh University, INDIA

## Abstract

This study investigates the impact of arc length, current intensity, travel speed, and gas flow rate on surface hardness and line width during arc quenching process of S45C steel. The current intensity has the greatest influence on the surface hardness of S45C steel, followed by the travel speed, gas flow rate, and arc length. Using the Taguchi method, the optimal values of the parameters such as the arc length of 1.5 mm, the current intensity of 125 A, the travel speed of 250 mm/min and the gas flow rate of 12.5 l/min were calculated. The optimal surface hardness would be 379 HV, with a standard deviation of 46.4 HV. The current intensity is the most critical component in determining line width among these parameters. The arc length ranks second, followed by the TIG gun’s travel speed. The gas flow rate is the least significant factor. A longer arc length may result in a broader heat zone, which leads to a better line width. Increasing the arc length, current intensity, travel speed, and gas flow rate results in a similar pattern of surface hardness change caused by the low-heated and over-heated phenomena. The microhardness distribution showed a hardening zone of up to 2500 μm and a maximum hardness of 453 HV. The microstructure of arc quenching samples has three zones: hardening, heat-affected, and base metal. The hardening zone exhibits a martensite microstructure with a tiny needle shape and a residual austenite matrix.

## Introduction

Heat treatment is a very important process of changing the properties of metallic materials used in the industrial field [1–3]. The microstructures and characteristics of heat-treated products can be modified for the desired purpose. The investigation into the effects of heat input on the microstructure, corrosion resistance, and mechanical properties of welded austenitic and duplex stainless steels was conducted by Mohammed, G.R., and colleagues [4]. According to the study’s findings, the amount of energy used during the welding process affects the ferrite-to-austenite ratio. Strengthening mechanisms and mechanical qualities are mostly dependent on the ferrite grain size, martensite volume fraction, and martensite morphology [5]. The effects of welding conditions and processes on the microstructure, mechanical characteristics, and corrosion resistance of duplex stainless steels and their various combinations based on the structure-property co-relationship are methodically highlighted in the research by Jagesvar Verma and Ravindra Vasantrao Taiwade [6]. Austenite is randomly dispersed in the ferritic matrix during intercritical annealing at slow heating rates. Conversely, excessive heating rates cause insufficient ferrite recrystallization, which produces zones with banded austenite [7]. The mechanical and technical qualities of the joint deteriorate as a result of the production of coarse grains and brittle morphologies in the heat-affected zone and weld metal [8]. According to Muhammad, M., and colleagues [9], duplex stainless steel [10] weld joints exhibit their best properties when the ferrite-austenite ratio is close to unity. It was discovered that there was a negative correlation between this ratio and corrosion current density and ultimate tensile strength and a positive correlation with hardness. A favorable correlation was also seen between the ferrite phase stabilizers and the final tensile strength. The microstructure, including the austenite, ferrite, and martensite phases, determines the parameters of the weld joint in martensitic stainless steel. Tensile strength and the ferrite phase were shown to be negatively associated, while hardness and impact toughness were positively correlated. The hardness and ultimate tensile strength were also found to be negatively correlated.

Heat treatment methods could be classified into two types, depending on how deep they affect. It includes volumetric heat treatment and surface heat treatment. Volumetric heat treatment has deeper effects and requires a whole volume heat-treating process, which costs time and energy. Surface heat treatment products, on the other hand, often have a thin layer of treatment on the surface. However, it can save time and energy when the heat-treated area only focuses on a limited area. Some popular surface physical heat treatment processes are induced quenching, laser quenching, flame quenching, electron beam quenching, and arc quenching [11–15].

Induced quenching is one of the methods of surface quenching, happening by induction-heating the surface of the magnetic materials following a quenching process [16–18]. This induced quenching has the advantages of rapid process, no decarburization, high hardness and wear resistance, and selective hardening. Javaheri *et al*. [19] investigated the effects of induction hardening of pipe steel on the grain size and slurry erosion performance. This study indicated that the martensite grain size strongly impacts the wear resistance and slurry erosion of the steel pipe. At a heating speed of 50°/s and a heating temperature of 1000°, the medium-carbon steel pipe could reach the extremely high hardness of 1270 Vickers Hardness (HV). Gao *et al*. [20] surveyed the artificial defects impacts on the fatigue strength of the S38C steel axles after induction hardening. The defects are created by the electrical discharge machining (EDM) method. The fatigue strength of EDM specimens decreases linearly as depth increases. Because of the presence of residual stress, the experimental findings exceed Murakami’s model prediction. Furthermore, fatigue cracks originated from secondary notches on the bottom of EDM flaws. Parvinzadeh *et al*. [21] wanted to reduce the edge effect during the induction hardening process of the 4340 steel spur gear. The control system contains two magnetic flux concentrations that are applied to modify the case depth and hardening layer profile of spur gears. To limit the edge effect and improve the case depth, the most important factors are heating time and the axial gap between concentrators and the spur gear. The lowest axial gap and longest heating time lead to the best quality with a low edge effect and good case depth.

Laser quenching applies a high-power diode laser to heat the metallic surface in an extremely short time [22–24]. The heated area is a selective region; it is heated to an austenite phase and then self-quenched to the martensite phase. Laser hardening has many merits: being suitable for complex shapes, high precision, low cost, and low rate of thermal distortion. Muthukumaran and Babu [25] examined the impact of different laser types, parameters, and steel grades on the characteristics of laser-hardening substrates. There are three zones in the heat-treated substrate, including the hardened zone, transition zone, and heat-affected zone. Changing laser types, parameters, and steel grades could obtain a case hardening depth of 0.15–2.3 mm. In particular, multiple quenching can cause annealing phenomena of tempered areas, which would decrease the hardness of the overlap areas upon laser treatment. Flame quenching uses a high-temperature flame generated from an oxygen-fuel flame to directly heat the steel or cast iron parts [26–28]. This quenching technique allows obtaining a layer with high wear resistance and hardness of 55‒60 HRC on a depth of 0.1 to 6.4 mm. Flame quenching also has the typical advantages of the surface hardening process, such as rapidity, high hardness, high wear resistance, and low distortion level. However, it also has some demerits of possible overheating, low rate of precision, oxidation, decarburization, and fire hazards. Electron beam quenching, similar to the laser quenching method, uses a high-energy source of electron beam to rapidly heat the workpiece to the austenite phase. After that, the surface is self-quenched by transferring the heat to the based materials. Thamilarasan *et al*. [29] reported the optimization of the flam hardening process by heating the low-carbon steel under an oxy-acetylene flame. The effects of flame temperature, torch cap, and quenching period on the hardness of the substrate are surveyed. The results find out that the quenching period is the most critical factor among them. The optimal parameters are a quenching period of 40 s, a flame temperature of 1000°C, and a torch cap of 35 mm. This parameter set could improve the steel hardness to 600 HV ‒ 700 HV. Arc quenching generates an arc between an electrode and a steel surface. Interestingly, Marichamy *et al*. [30] also optimized the flame hardening process of Eglin steel by using Taguchi methods. The Eglin steel is heated by an oxy-acetylene flame. The optimal parameters for the highest hardness are surface temperature of 1000°, torch cap of 40 mm, and quenching period of 40 s. The surface temperature before quenching is the most important factor that could strongly affect the hardness of the Eglin steel.

Compared to laser quenching and electron beam quenching, arc generation equipment is cheaper, more simple, and more available [31]. Safonov *et al*. [32] reported that the maximum hardness of this arc quenching technique could reach 50–60 HRC with a maximum case depth of 1.5–2.0 mm, depending on the chemical composition of the sample. Mikheev *et al*. [33] reveal that applying the arc quenching method could improve the wear resistance of the medium-carbon steel four times. The surface microhardness of the 0.4% carbon steel could obtain a high value of 7.5 GPa. However, the investigation about arc quenching is rarely conducted. The influence of arc quenching parameters such as arc length, current intensity, travel speed, and gas flow rate needs more reports to shed more light on this technique.

This study tried to survey the impact of arc quenching parameters on the properties of carbon steel. The impacts of arc length, current intensity, travel speed, and gas flow rate on the hardness of the carbon steel are examined. The microstructure of the arc quenching sample is also investigated. The arc quenching equipment is a combination of a tungsten inner gas (TIG) machine and a computer numerical control (CNC) machine, which is available in the industry. The study results could provide more information about this heat treatment technique and improve the application of this technique in the industry field.

## Experimental methods

**Fig 1:**
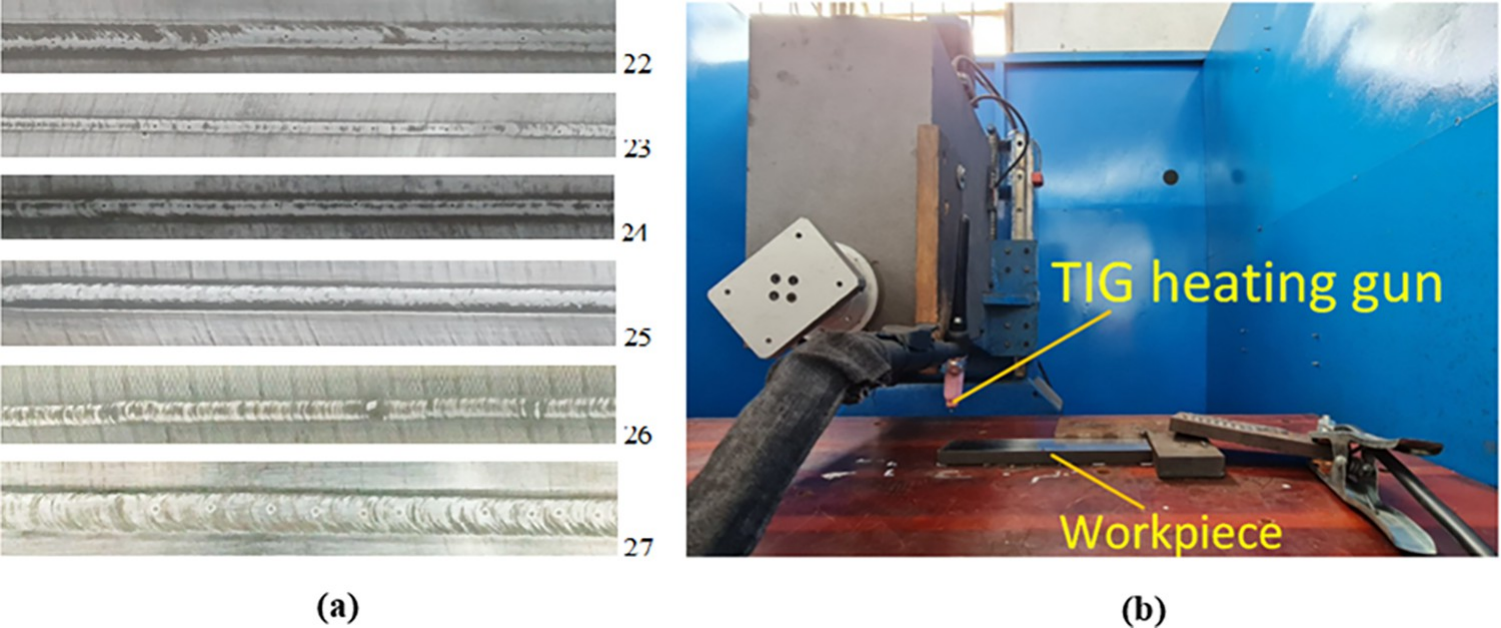
Hardening surfaces and arc quenching process. (a) Hardening surfaces, and (b) arc quenching machine.

This study uses S45C plain carbon steel with the nominal chemical composition shown in Table 1. The steel plate has the rectangular shape and the dimensions of 200 mm x 40 mm x 15 mm. Fig 1 shows the arc quenching surface, and arc quenching machine. The arc quenching machine is the assembly of TIG and a CNC machine. The surveyed parameters during the arc quenching process were shown in Table 2. First, the arc length from the torch to the surface or arc gap is the Z variable along with three levels: 0.5 mm, 1 mm, and 1.5 mm. Next, the I variable is the current intensity with three levels: 50 A, 125 A, and 200 A. The V variable is the travel rate with three levels: 80 mm/min, 165 mm/min, and 250 mm/min. Finally, the G variable is the gas flow rate with three levels: 5 l/min, 12.5 l/min, and 20 l/min. These parameters are shown in Table 3. This table was designed by the Taguchi method using Minitab 19.1 software. The durable design method, also referred to as the Taguchi approach, is a useful tactic for improving products [34, 35]. By identifying the ideal process parameters, the Taguchi technique makes use of statistics to enhance the quality of the result [36]. To ascertain the best arrangement of components and their respective levels for generating sample sets, researchers utilized the Taguchi design of trials. By using orthogonal arrays, the Taguchi technique has the advantage of requiring fewer experiments or simulations and minimizing the impact of uncontrollable parameters [37]. It’s also an easy-to-use and basic method. As a result, the author decided to base this analysis on the orthogonal table of 27 examples (Table 3).

**Table 1:** Chemical composition of S45C steel for the arc quenching process.

Weight %	C	Si	Mn	P	S	Ni	Cr
S45C	0.42–0.50	0.17–0.37	0.5–0.8	0.035 max	0.035 max	0.25 max	0.25 max

**Table 2:** The factors and their levels.

Design variables	Symbol	Unit	Level 1	Level 2	Level 3
The arc length from the torch to the surface or arc gap	Z	mm	0.5	1	1.5
The current intensity	I	A	50	125	200
The travel rate of the TIG gun	V	mm/min	80	165	250
The Argon gas flow rate	G	l/min	5	12.5	20

**Table 3:** Surface hardness and line width of Taguchi designed samples.

Sample No.	Z	I	V	G	Average surface hardness (HV)	Line width (mm)
	(mm)	(A)	(mm/min)	(l/min)		
1	0.5	50	80	5.0	250	3.6
2	0.5	50	165	12.5	305	2.3
3	0.5	50	250	20	249	3.1
4	0.5	125	80	5.0	296	4.2
5	0.5	125	165	12.5	366	4.1
6	0.5	125	250	20	418	2.1
7	0.5	200	80	5.0	209	5.5
8	0.5	200	165	12.5	385	3.5
9	0.5	200	250	20	376	4.1
10	1.0	50	80	5.0	312	3.6
11	1.0	50	165	12.5	304	2.5
12	1.0	50	250	20	346	3.2
13	1.0	125	80	5.0	334	3.6
14	1.0	125	165	12.5	307	3.3
15	1.0	125	250	20	376	3.3
16	1.0	200	80	5.0	215	5.7
17	1.0	200	165	12.5	333	5.1
18	1.0	200	250	20	389	5.5
19	1.5	50	80	5.0	344	3.6
20	1.5	50	165	12.5	365	2.5
21	1.5	50	250	20	336	3.4
22	1.5	125	80	5.0	289	6.5
23	1.5	125	165	12.5	409	4.6
24	1.5	125	250	20	327	3.6
25	1.5	200	80	5.0	387	4.7
26	1.5	200	165	12.5	286	4.9
27	1.5	200	250	20	298	5.6

After the arc quenching process, the hardened substrates are evaluated via a hardness test and a microstructure test. The hardness test is conducted by the HR-150A Rockwell hardness tester, Yisite, Shenzhen, China. The hardness test process was carried out at 10 points in the samples (S1–S5 Figs). The average value of the 10 hardness measurement points of each test sample was the hardness value for that test sample (S1 Table). The microhardness is tested via Vickers hardness tester [38] (HV) HM101 Mitutoyo, Tokyo, Japan. The value of the indentation was performed by applying a load of 2.942 N for 15 seconds when we used to measure hardness with the Vickers method. The microhardness test position of the sample was measured at the center position of the quenching zone and measurements were performed at 25 depth positions of the test sample (S6 Fig). The width of the hardened surface was also measured using a digital caliper (Mitutoyo 500-703-20, Tokyo, Japan). The samples are evaluated for metallurgical microstructure after being polished and etched with 4% Nital solution. The optical microscope named Oxion OX.2153-PLM EUROMEX, Holland, is used to obtain the microstructure of the quenched samples. Moreover, the sample surfaces are also observed via a scanning electron microscope (SEM) named JEOL 5410 LV, Japan.

## Results and discussion

As mentioned above, the experiment samples are designed via the Taguchi method. The surface hardness and line width of the arc quenching samples are presented in Table 3. From this table result, other figures and tables can be calculated to reveal the importance level of these factors and the optimization parameters. Remarkably, all surface hardness values are greatly higher than the initial hardness before arc quenching, which is only 139 HV, indicating the effectiveness of the arc quenching process.

**Table 3** shows that the highest Surface hardness in sample 6 is 418 HV and sample 16 has the smallest Surface hardness of 215 HV. For Line width, case 22 has the largest Line width of 6.5 mm, case 6 has the smallest Line width of 2.1mm. Also in this table, looking at the overview, we can see the contrast between Surface hardness and Line width. When the Surface hardness has the largest value, the Line width has the smallest value. This can be explained that when the heating is concentrated at a small point, the surface will receive more heat and the quenching process will be more effective when the same amount of heat is dispersed over a larger width, which will receive less heat.

Table 4 shows the Response Table for Means of the S45C steel surface hardness with larger is better criteria. The results indicate that the current intensity has the highest impact level on the surface hardness of the S45C steel, followed by the travel speed. The gas flow rate has the third position, while the arc length has the lowest impact level on the surface hardness. It means that controlling the current intensity and travel speed has a higher efficiency than the gas flow rate and arc length parameters. The reasons for this order are that the heat input of the arc gun is the ratio to the current intensity and the inverse ratio to the travel speed [39].

**Table 4:** Response table for means of the S45C steel surface hardness (larger is better).

Level	Z	I	V	G
1	315.7	309.6	304.6	308.2
2	321.3	343.6	332.2	337.6
3	334.7	318.6	334.9	325.9
Delta	19.0	34.0	30.3	29.3
Rank	4	1	2	3

**Fig 2:**
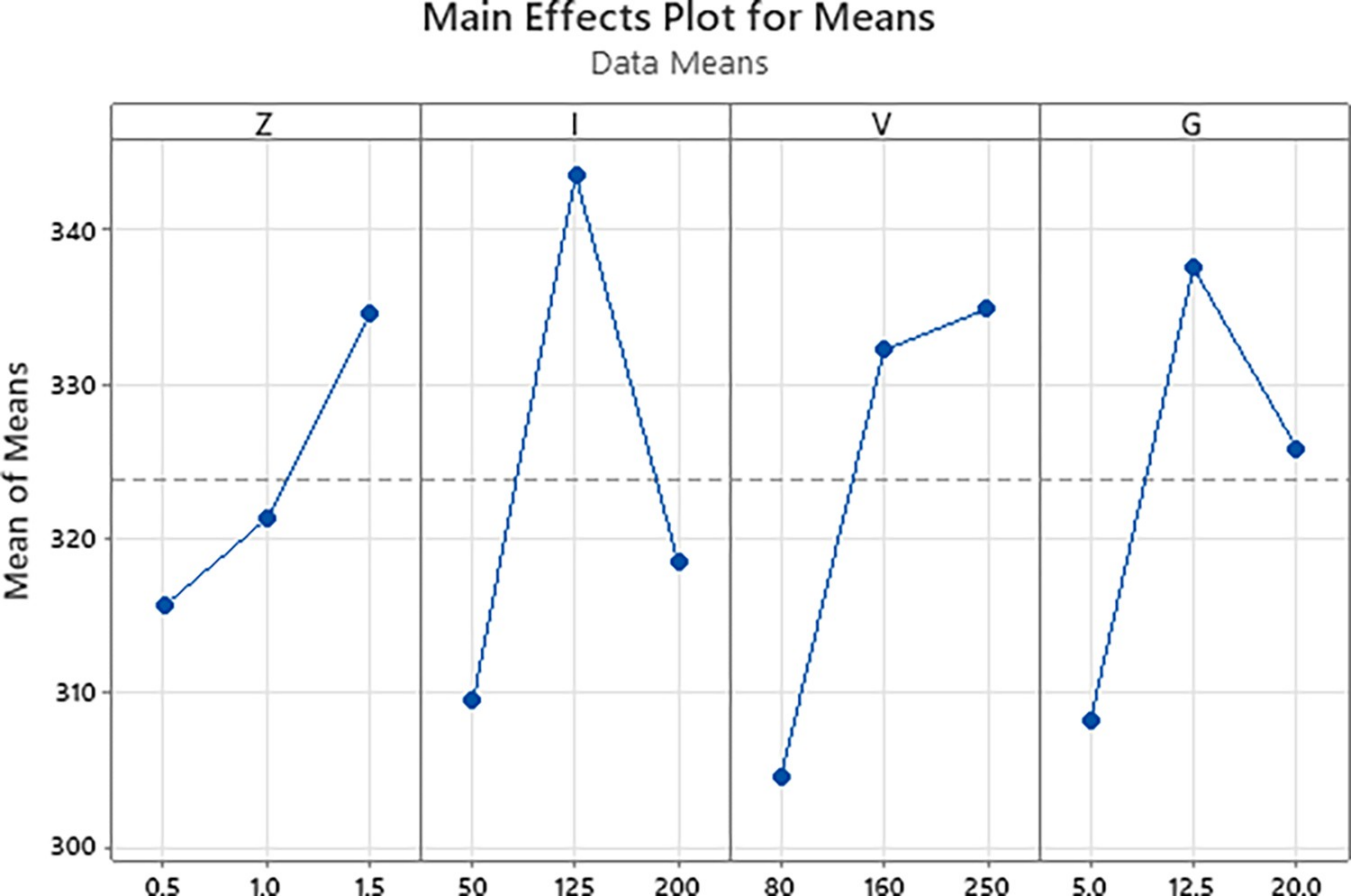
Main effects plot for means of the S45C steel surface hardness (larger is better).

Fig 2 shows the Main effects plot for Means of the S45C steel surface hardness with larger is better criteria. The optimal parameters that can be withdrawn from this figure are arc length of 1.5 mm, current intensity of 125 A, travel speed of 250 mm/min, and gas flow rate of 12.5 l/min. The predicted surface hardness calculated from this parameter set is 379 HV with a standard deviation of 46.4 HV.

If the TIG gun is too close to the steel substrate, the heat could be too high, leading to the melting phenomenon, which is similar to the welding process [40]. Therefore, a higher level of arc length could avoid the negative effect of the overheating phenomenon, leading to better surface hardness.

Table 5 shows the Response Table for Means of the S45C steel quenched line width with larger is better criteria. Similar to the surface hardness results, this table also shows that the current intensity plays the most important factor among these parameters. The arc length ranks in the second position, followed by the travel speed of the TIG gun. Because a higher arc length could lead to a wider heat zone and a better line width [41]. The gas flow rate is the least important factor. Therefore, focusing on controlling the current intensity and arc length could achieve the desired line width.

**Table 5:** Response table for means of the S45C steel quenched line width (larger is better).

Level	Z	I	V	G
1	3.578	3.022	4.367	3.767
2	3.889	3.911	3.833	4.011
3	4.367	4.900	3.633	4.056
Delta	0.789	1.878	0.733	0.289
Rank	2	1	3	4

**Fig 3:**
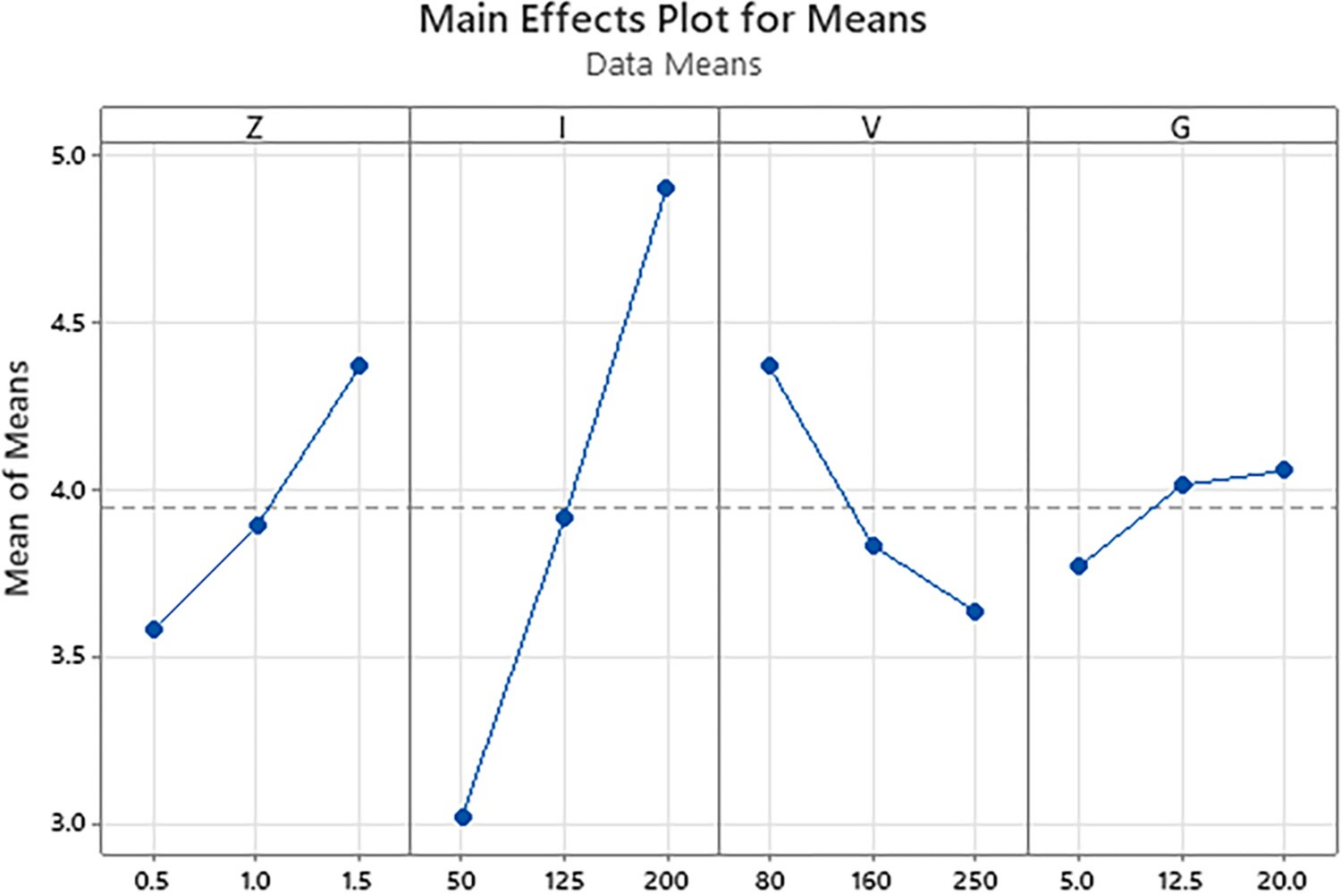
Main effects plot for means of the S45C steel quenched line width (larger is better).

Fig 3 shows the Main effects plot for Means of the S45C steel quenched line width with larger is better criteria. The results show that the optimal parameters are arc length of 1.5 mm, current intensity of 200 A, travel speed of 80 mm/min, and gas flow rate of 20 l/min. The predicted line width calculated from this parameter set is 5.86 mm with a standard deviation of 1.19 mm.

On the other hand, the current intensity, travel speed, and gas flow rate have a significantly lower ratio than the arc length factor. Because a longer arc length may result in a broader heat zone, which leads to a better line width.

Besides the Taguchi design, this report also investigates the surface hardness in the more traditional method, which is a univariate method. Table 6 shows the measurement data of experimental samples by using the classic, alternative method. These parameters are selected differently from the Taguchi method, with a smaller division to further investigate each parameter. The results from this table are analyzed via the following figures.

**Table 6:** Measurement data of the surface hardness by using univariate method.

Sample	Z	I	V	G	Average surface hardness (HV)
	(mm)	(A)	(mm/min)	(l/min)	
1	0.5	50	80	5	249
2	1				309
3	1.5				389
4	2				366
5	2.5				288
6	1.5	75			296
7		100			408
8		125			366
9		150			306
10		175			301
11		100	100		332
12			120		364
13			140		386
14			160		373
15			180		364
16			120	8	295
17				11	387
18				14	332
19				17	304
20				20	302

**Fig 4:**
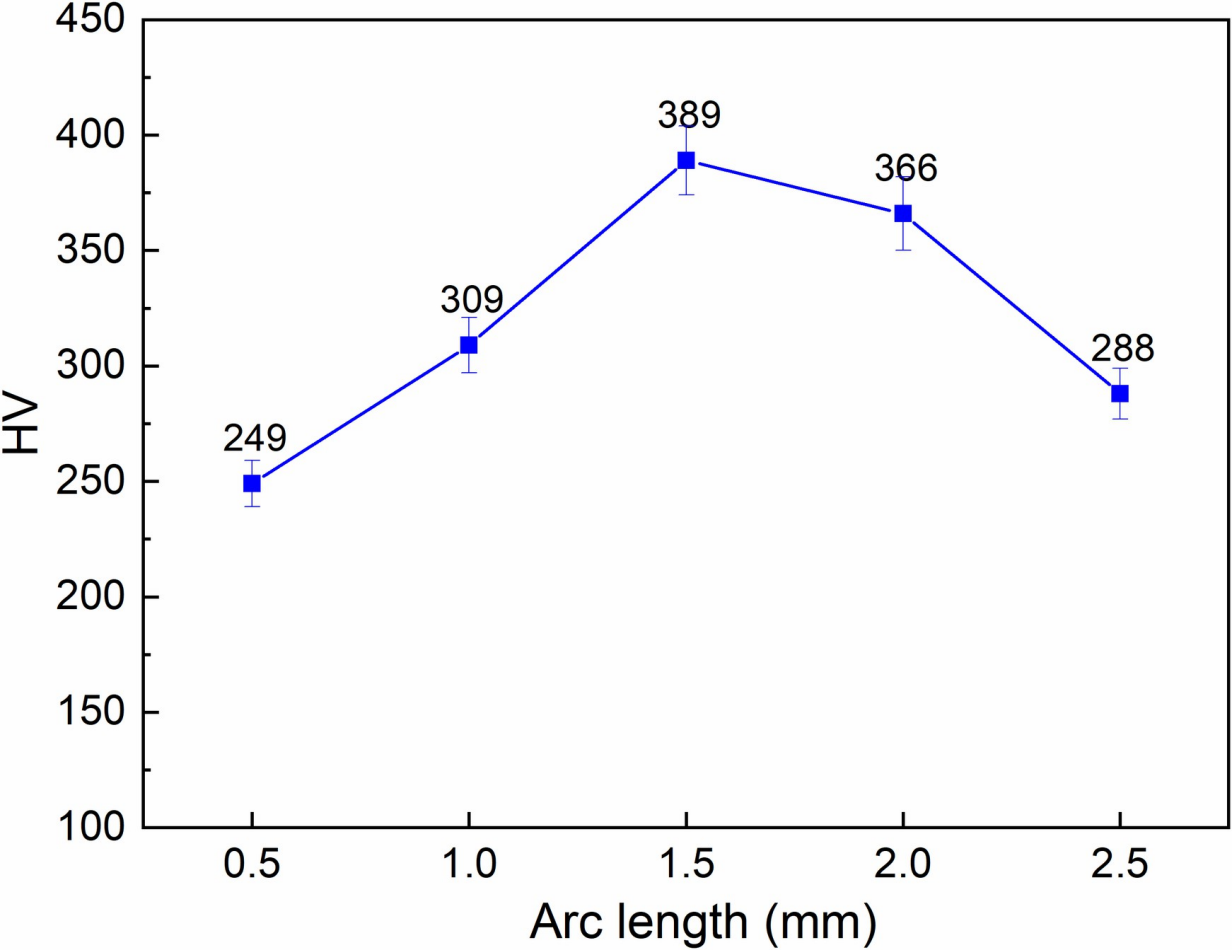
Relationship between arc length and surface hardness of S45C steel after arc quenching.

Fig 4 depicts the relationship between arc length and surface hardness of S45C steel after arc quenching. The current intensity is set at 50 A, the travel speed is 80 mm/min, and the gas flow rate is 5 l/min. The surface hardness values are 249 HV, 309 HV, 389 HV, 366 HV, and 288 HV corresponding to the arc length of 0.5 mm, 1.0 mm, 1.5 mm, 2.0 mm, and 2.5 mm. The maximum hardness value is 389 HV with an arc length of 1.5 mm, while the minimum hardness value is 249HV with an arc length of 0.5 mm. Interestingly, the surface hardness of the S45C steel could be divided into two stages. Firstly, from 0.5 mm to 1.5 mm, an increase in the arc length leads to a higher level of surface hardness. The reason for this phenomenon is above mentioned. Increasing the arc length in this rank will lead to a reduction in the overheated effect; therefore, the surface hardness is improved. Secondly, from 1.5 mm to 2.5 mm, the surface hardness value declines due to the lower temperature of the steel surface [42]. If the temperature of the surface is lower than the full austenite temperature range, the hardness is declined.

**Fig 5:**
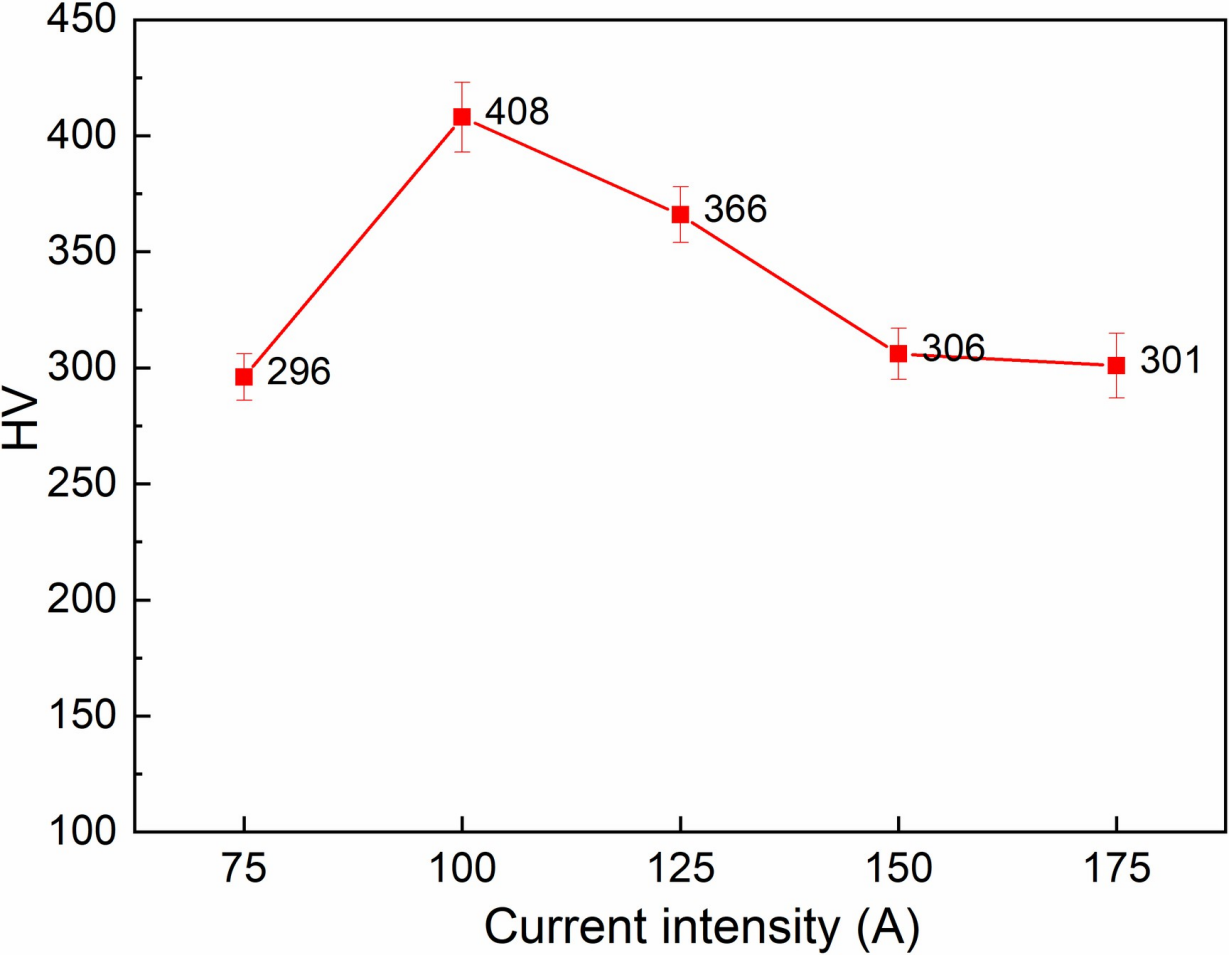
Relationship between current intensity and surface hardness of S45C steel after arc quenching.

Fig 5 presents the relationship between current intensity and surface hardness of S45C steel after arc quenching. The arc length is set at 1.5 mm, the travel speed is 80 mm/min, and the gas flow rate is 5 l/min. The surface hardness values are 296 HV, 408 HV, 366 HV, 306 HV, and 301 HV, corresponding to the current intensity of 75 A, 100 A, 125 A, 150 A, and 175 A. At 100 A, the surface hardness gains its maximum value of 408 HV, while the lowest hardness value is 296 HV at the current intensity of 75 A. Remarkably, the changing pattern of this figure is similar to the relationship between the arc length and the surface hardness. In this trend, the overheating of the S45C steel could happen when the intensity is higher than 100 A. Because too high current intensity leads to the generation of too much heat [43, 44]. On the other hand, the current intensity, which is lower than 100 A does not generate enough heat to achieve a fully austenite temperature range. This is the reason for the lower surface hardness when quenching at 75 A.

**Fig 6:**
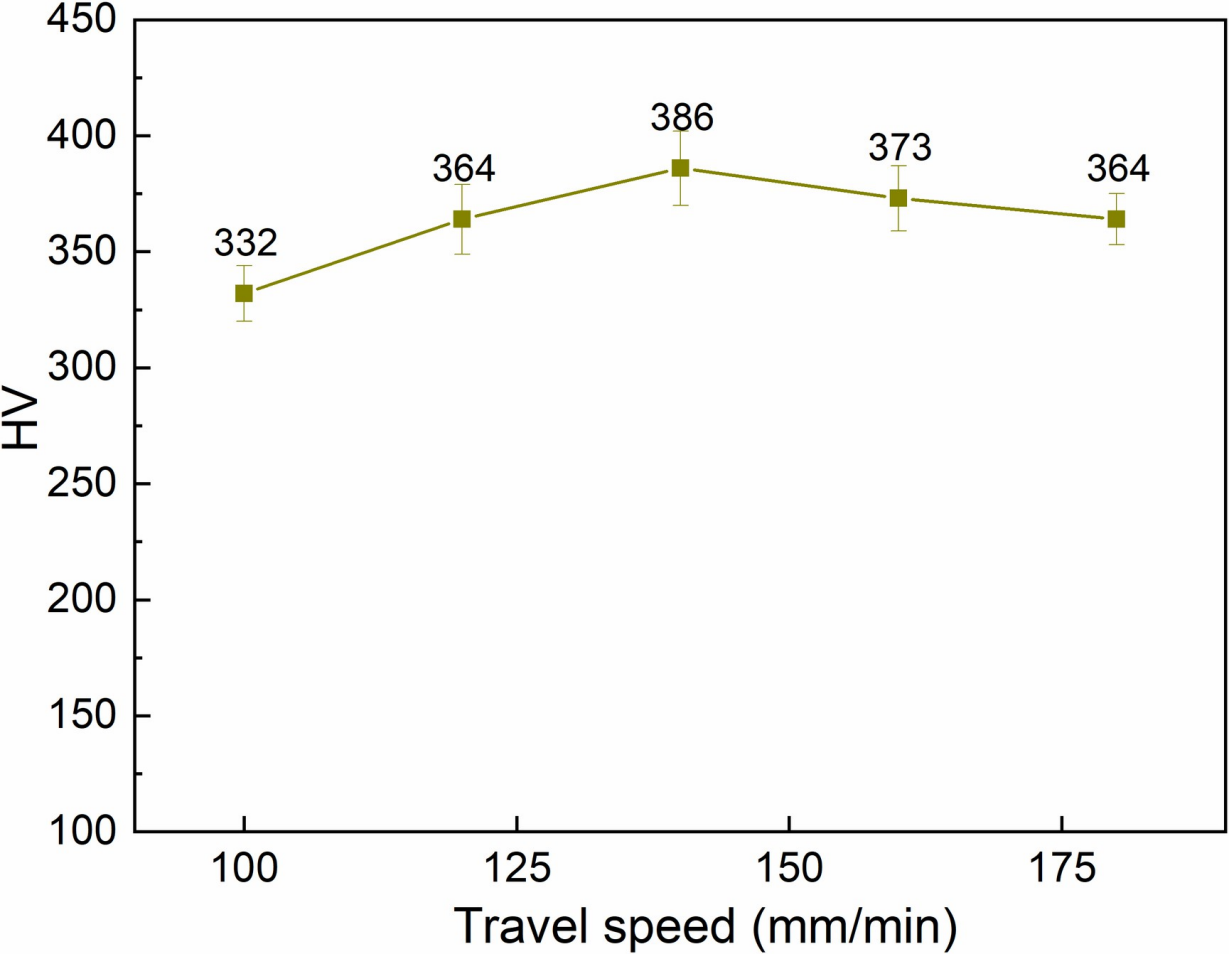
Relationship between travel speed and surface hardness of S45C steel after arc quenching.

The relationship between the travel speed and surface hardness of S45C steel after arc quenching is presented in Fig 6. The arc length is set at 1.5 mm, the current intensity is 100 A, and the gas flow rate is 5 l/min. The results reveal a similar changing pattern to the arc length and the current intensity parameters with a two-stage phenomenon. At the first stage of 100 mm/min ‒140 mm/min, an increase in the travel speed leads to the gradual improvement of the S45C surface hardness from 332 HV to 386 HV. The reason is that the overheated phenomenon of the low travel speed is gradually reduced when the travel speed increases. It means that the low travel speed leads to the heat concentrating on the quenched line, leading to the overheating issue [45]. In reverse, at the second stage of 140 mm/min ‒ 180 mm/min, the S45C steel surface does not achieve a temperature high enough to have the full austenite phase. Therefore, it leads to a reduction in the surface hardness.

**Fig 7:**
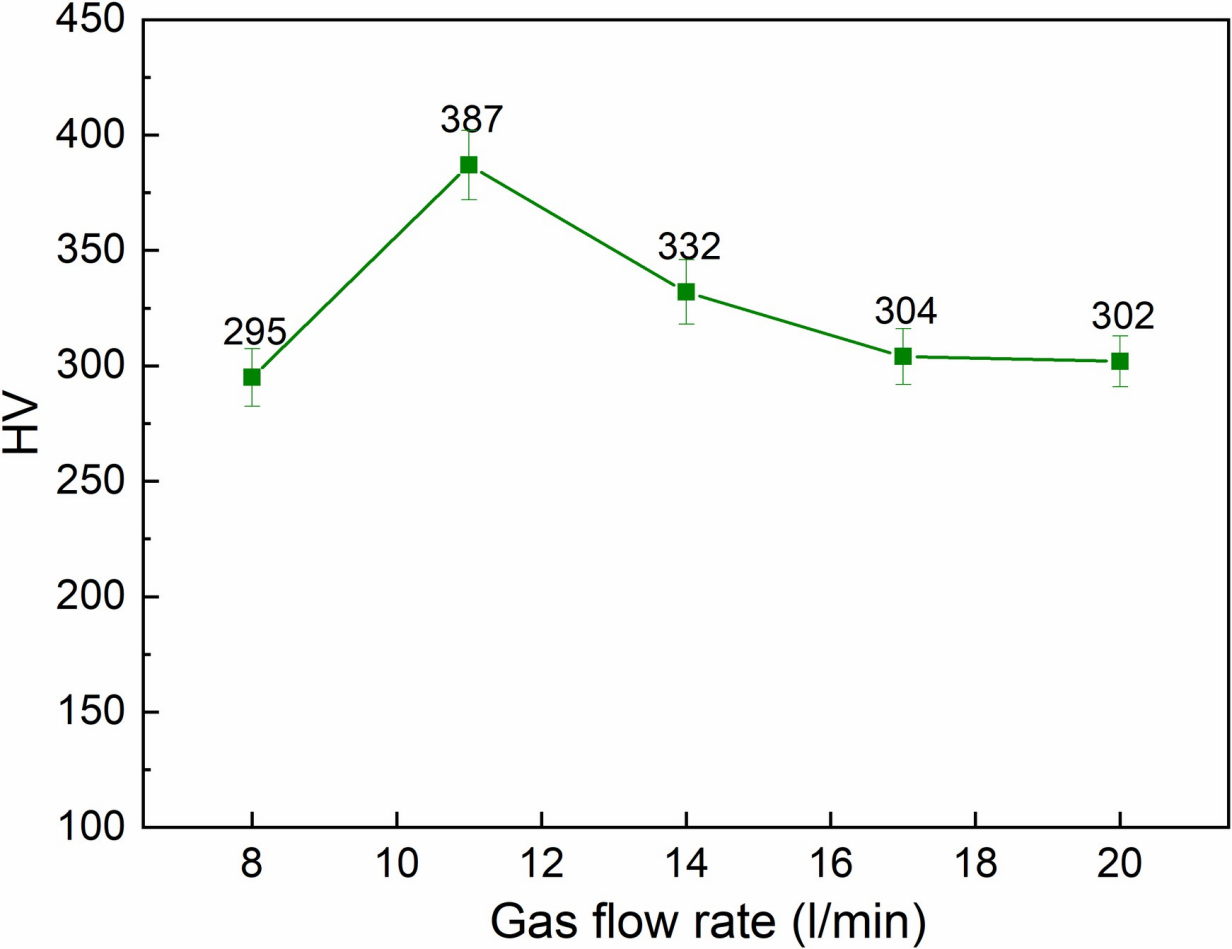
Relationship between gas flow rate and surface hardness of S45C steel after arc quenching.

Fig 7 shows the relationship between gas flow rate and surface hardness of S45C steel after arc quenching. The arc length is set at 1.5 mm, the current intensity is 100 A, and the travel speed is 120 mm/min. A similar pattern of surface hardness changes when the gas flow rate is improved from 8 l/min to 20 l/min. At the first stage of 8 l/min ‒11 l/min, an increase in the Argon gas flow rate leads to the gradual improvement of the S45C surface hardness from 295 HV to 387 HV. In the second stage of 11 l/min ‒20 l/min, an increase in the Argon gas flow rate reduces the hardness value of the S45C surface from 387 HV to 302 HV. This change is similar to the analysis using the Taguchi method.

**Fig 8:**
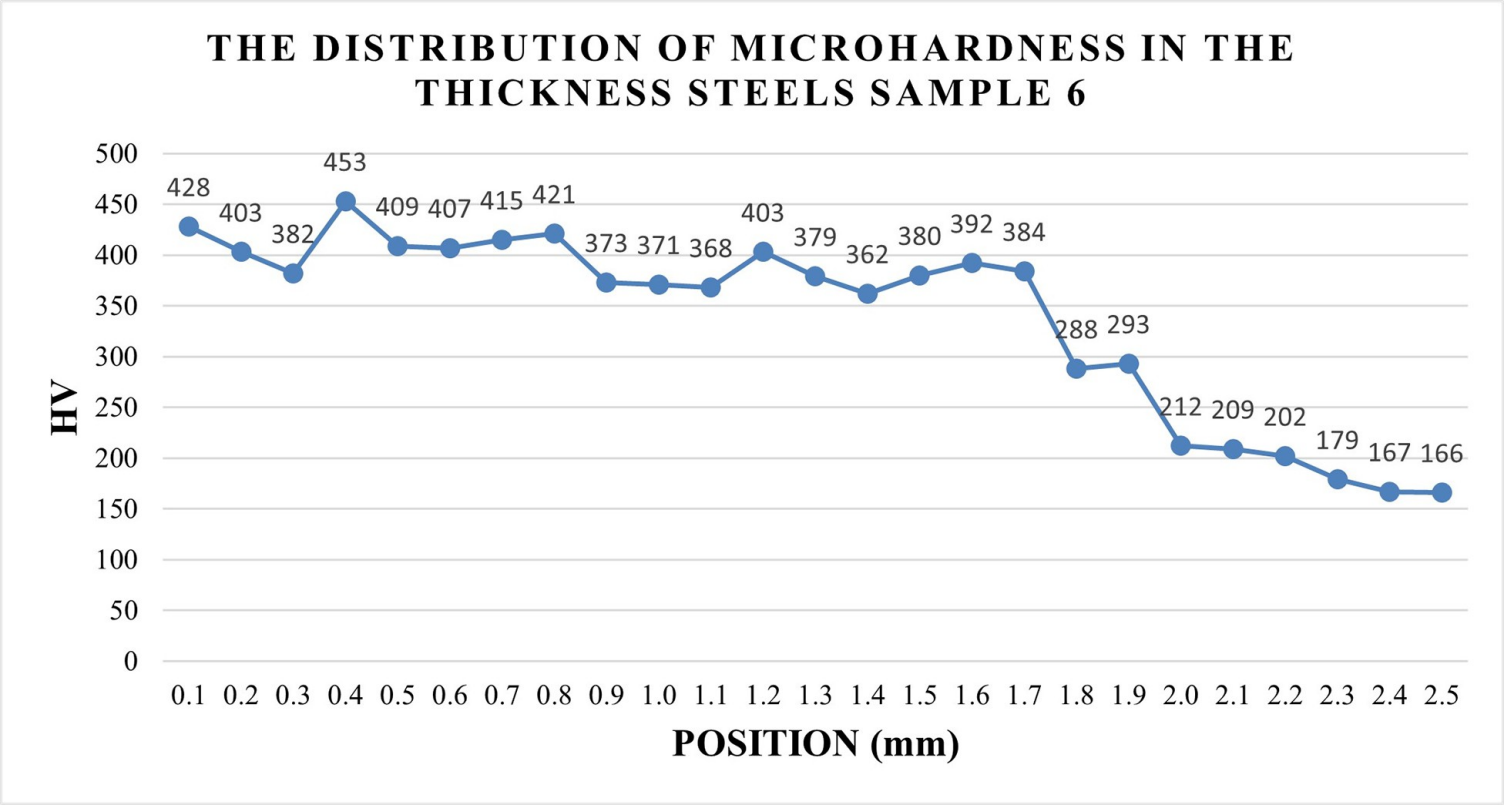
Distribution of microhardness in the cross-section of S45C steels sample No.6.

Fig 8 displays the distribution of microhardness in the cross-section of S45C steel sample No.6 from Table 3, which has the highest hardness value among other samples in this table. The distribution of microhardness mostly shows a consistent decreasing trend from above 400 HV at the outer surface to 167 HV at the 2500 μm position from the surface. The highest microhardness value of sample 6 is 453 HV at a position of 400 μm, which is 3.3 times higher than the initial hardness of 139 HV. The lowest microhardness value is 166 HV at a position of 2500 μm, which is 1.2 times higher than the initial hardness of 139 HV, indicating that the case depth could be higher than 2500 μm. The distance from 0 μm to 1700 μm represents the hardening zone. While the distance from 1700 μm to 2500 μm represents the position of the heat-affected zone. Over 2500 μm is the base metal that is not affected by the heat. In general, the high-hardness zone is from the surface to the boundary between the hardening zone and the heat-affected zone.

**Fig 9:**
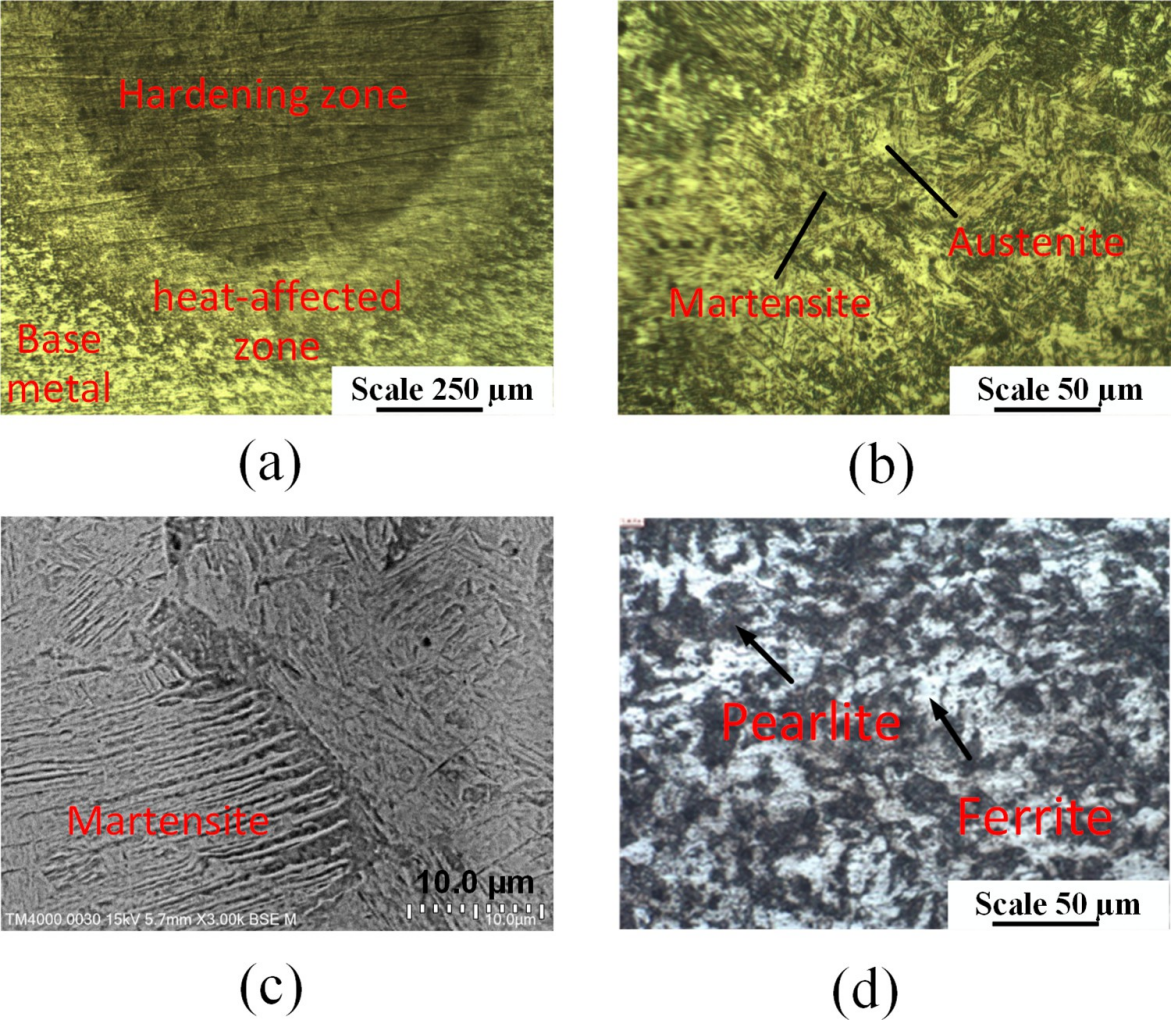
Microstructure of the S45C steel sample after arc quenching. (a) overview of the quenched area (S7 Fig), (b) and (c) Martensite and austenite structures of the hardening zone (S8 and S9 Figs), (d) perlitic and ferritic phases of the base metal.

Fig 9 shows the microstructure of the S45C steel sample after arc quenching. Fig 9(A) shows the overview of the microstructure across 3 zones, including the hardening zone, heat-affected zone, and base metal. Microhardness distribution in Fig 8 presents the gradual reduction of hardness from the hardening zone, heat-affected zone, and base metal, corresponding to the microstructure of martensite and residual austenite, and the ferrite and pearlite phases. Fig 9(B) and 9(C) reveals the microstructure of the martensite phase with a fine needle shape and the residual austenite matrix of the hardened zone, a similar result to Mikheev *et al*. [33] report. These microstructures prove that the S45C samples are successfully quenched by the arc quenching method. This zone has a curved shape with the center being on the surface, indicating the heat energy transfer of the arc. In addition, the microstructure of the base metal zone, as shown in Fig 9(D), shows the ferrite and pearlite of the S45C steel.

## Conclusion

In this study, the arc quenching of the S45C steel is investigated by examining the impact of arc length, current intensity, travel speed, and gas flow rate on the surface hardness and line width. Some important results could be revealed are:

- With the surface hardness, the current intensity has the highest impact level on the surface hardness of the S45C steel, followed by the travel speed, the gas flow rate, and the arc length. If the TIG gun is too close to the steel substrate, the heat could be too high, leading to the melting phenomenon and a reduction in the surface hardness. The optimal parameters that calculated from Taguchi method are arc length of 1.5 mm, current intensity of 125 A, travel speed of 250 mm/min, and gas flow rate of 12.5 l/min. The optimal surface hardness could be 379 HV with standard deviation of 46.4 HV.
- With the line width, the current intensity plays the most important factor on the line width among these parameters. The arc length ranks the second position, following by the travel speed of the TIG gun. The gas flow rate is the least important factor. Higher arc length could lead to a wider heat zone, leading to a better line width. The optimal parameters are arc length of 1.5 mm, current intensity of 200 A, travel speed of 80 mm/min, and gas flow rate of 20 l/min. The optimal line width calculated from this parameter set are 5.86 mm with standard deviation of 1.19 mm.
- Increasing the arc length, current intensity, travel speed, and gas flow rate have a similar pattern of surface hardness changing due to the low-heated and over-heated phenomena. The microhardness distribution indicated that the hardening zone could reach to 2500 μm with the highest hardness of 453 HV. The microstructure of the arc quenching samples can be divided into three zone: hardening zone, heat-affected zone, and base metal. The hardening zone has the microstructure of martensite phase with a fine needle shape and the residual austenite matrix.

## Supporting information

S1 Fig The hardness measurement positions of samples 1–6. Hardness measurement positions were marked with red squares and measurements were conducted on the HR-150A Rockwell hardness tester.
(TIF)

S2 Fig The hardness measurement positions of samples 7–12. Hardness measurement positions were marked with red squares and measurements were conducted on the HR-150A Rockwell hardness tester.
(TIF)

S3 Fig The hardness measurement positions of samples 13–18. Hardness measurement positions were marked with red squares and measurements were conducted on the HR-150A Rockwell hardness tester.
(TIF)

S4 Fig The hardness measurement positions of samples 19–24. Hardness measurement positions were marked with red squares and measurements were conducted on the HR-150A Rockwell hardness tester.
(TIF)

S5 Fig The hardness measurement positions of samples 25–27. Hardness measurement positions were marked with red squares and measurements were conducted on the HR-150A Rockwell hardness tester.
(TIF)

S6 Fig The microhardness measurement positions of sample 6. Hardness measurement positions were marked with red squares and measurements were conducted on the Vickers hardness tester.
(TIF)

S7 Fig Overview of the quenched area. After quenching, the samples were polished and etched with 4% Nital solution. The microstructure of the quenched samples were obtained by the optical microscope named Oxion OX.2153-PLM EUROMEX, Holland.
(TIF)

S8 Fig Martensite and austenite structures of the hardening zone. They were obtained by the optical microscope named Oxion OX.2153-PLM EUROMEX, Holland.
(TIF)

S9 Fig The martensite microstructure has a needle-like shape. It was also observed via a scanning electron microscope (SEM) named JEOL 5410 LV, Japan.
(TIF)

S1 Table Hardness parameters of 27 samples after electric arc quenching. The hardness measurements were conducted on the HR-150A Rockwell hardness tester.
(XLSX)
