# Supplementary figures and images for: Optimization of arc quenching parameters for enhancing surface hardness and line width in S45C steel using Taguchi method
Source: PLoS One. 2024 Dec 2;19(12):e0314648. doi: 10.1371/journal.pone.0314648 (PMC11611132; doi:10.1371/journal.pone.0314648)

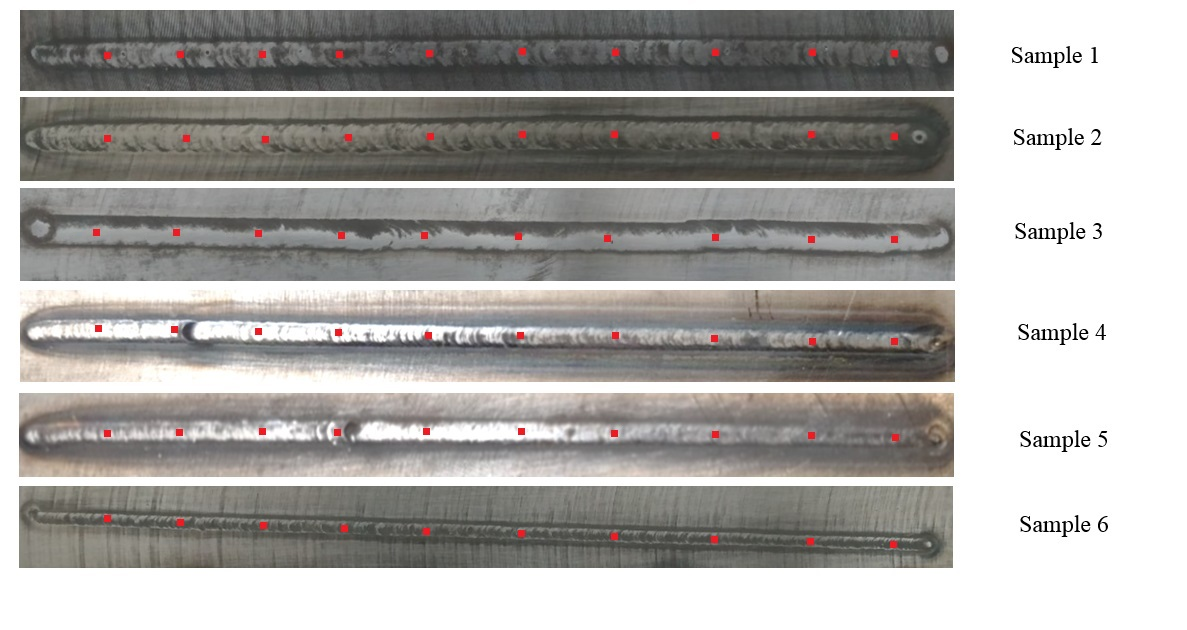

Supplement: S1 Fig — Hardness measurement positions were marked with red squares and measurements were conducted on the HR-150A Rockwell hardness tester. (TIF) [file pone.0314648.s001.tif]

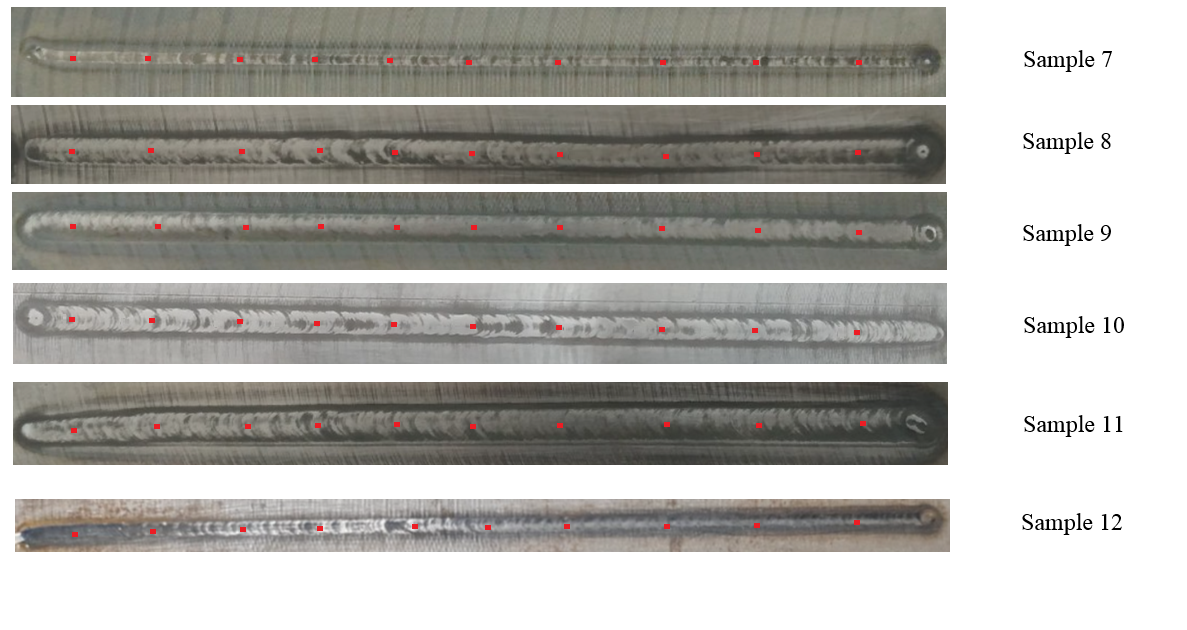

Supplement: S2 Fig — Hardness measurement positions were marked with red squares and measurements were conducted on the HR-150A Rockwell hardness tester. (TIF) [file pone.0314648.s002.tif]

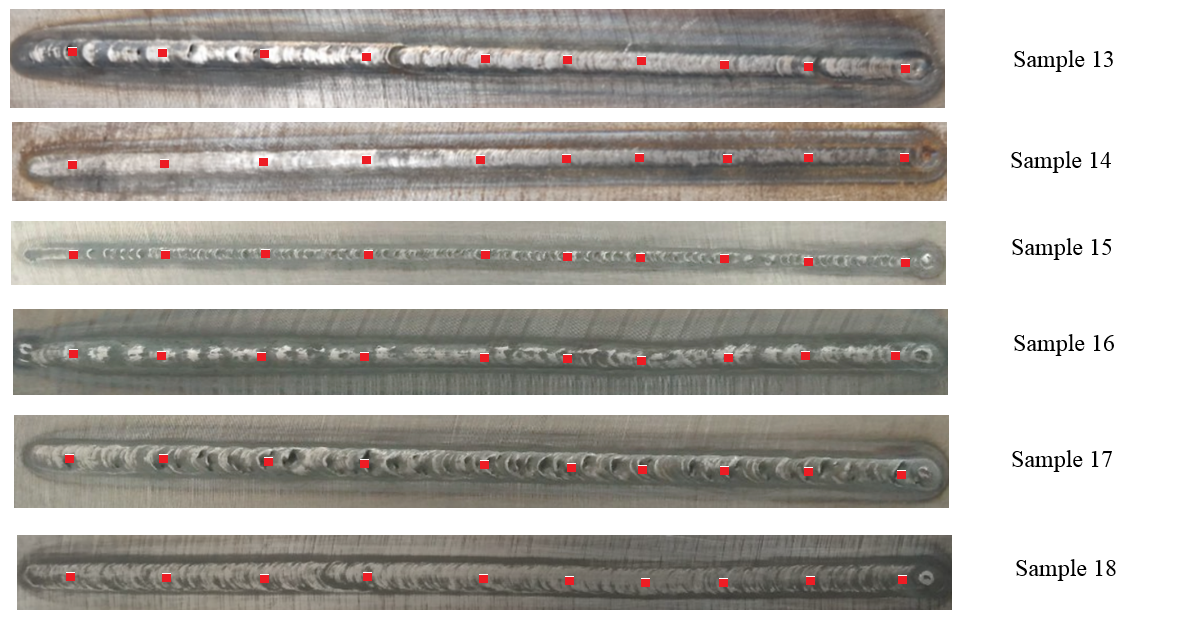

Supplement: S3 Fig — Hardness measurement positions were marked with red squares and measurements were conducted on the HR-150A Rockwell hardness tester. (TIF) [file pone.0314648.s003.tif]

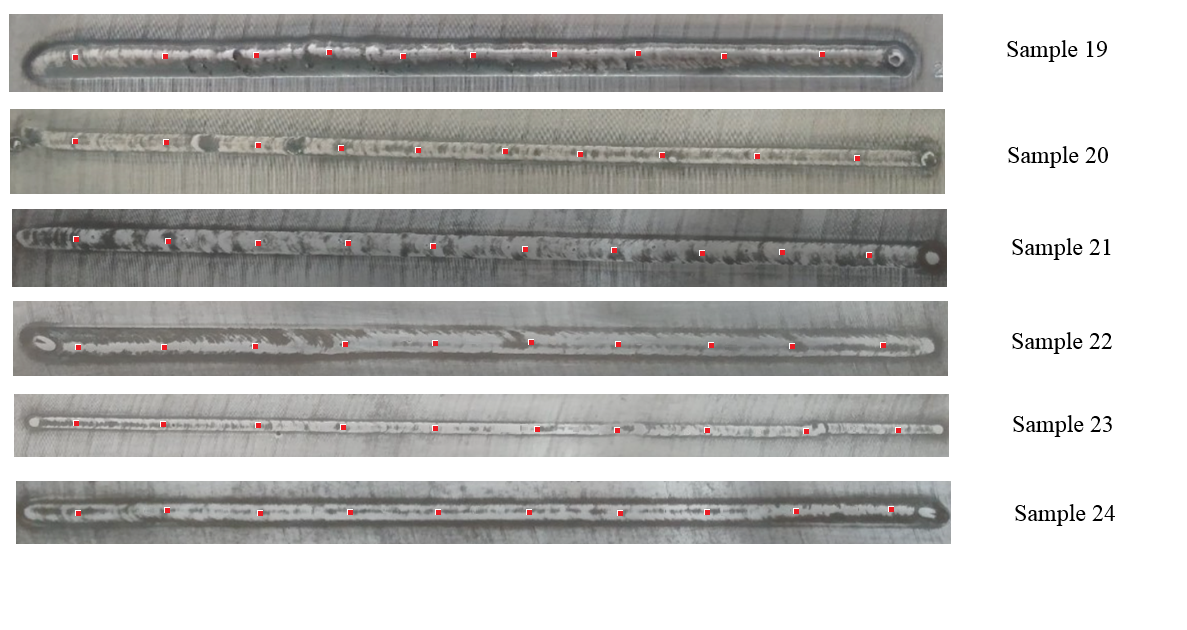

Supplement: S4 Fig — Hardness measurement positions were marked with red squares and measurements were conducted on the HR-150A Rockwell hardness tester. (TIF) [file pone.0314648.s004.tif]

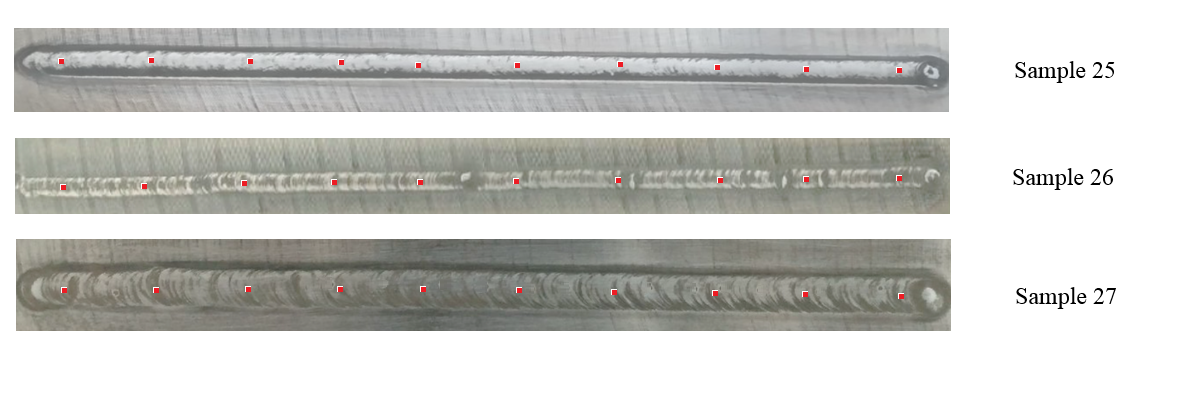

Supplement: S5 Fig — Hardness measurement positions were marked with red squares and measurements were conducted on the HR-150A Rockwell hardness tester. (TIF) [file pone.0314648.s005.tif]

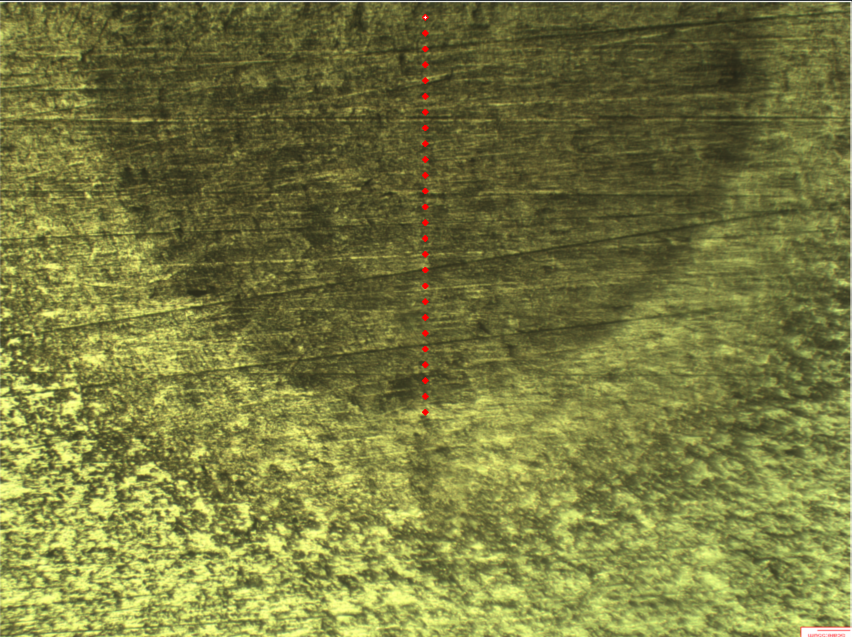

Supplement: S6 Fig — Hardness measurement positions were marked with red squares and measurements were conducted on the Vickers hardness tester. (TIF) [file pone.0314648.s006.tif]

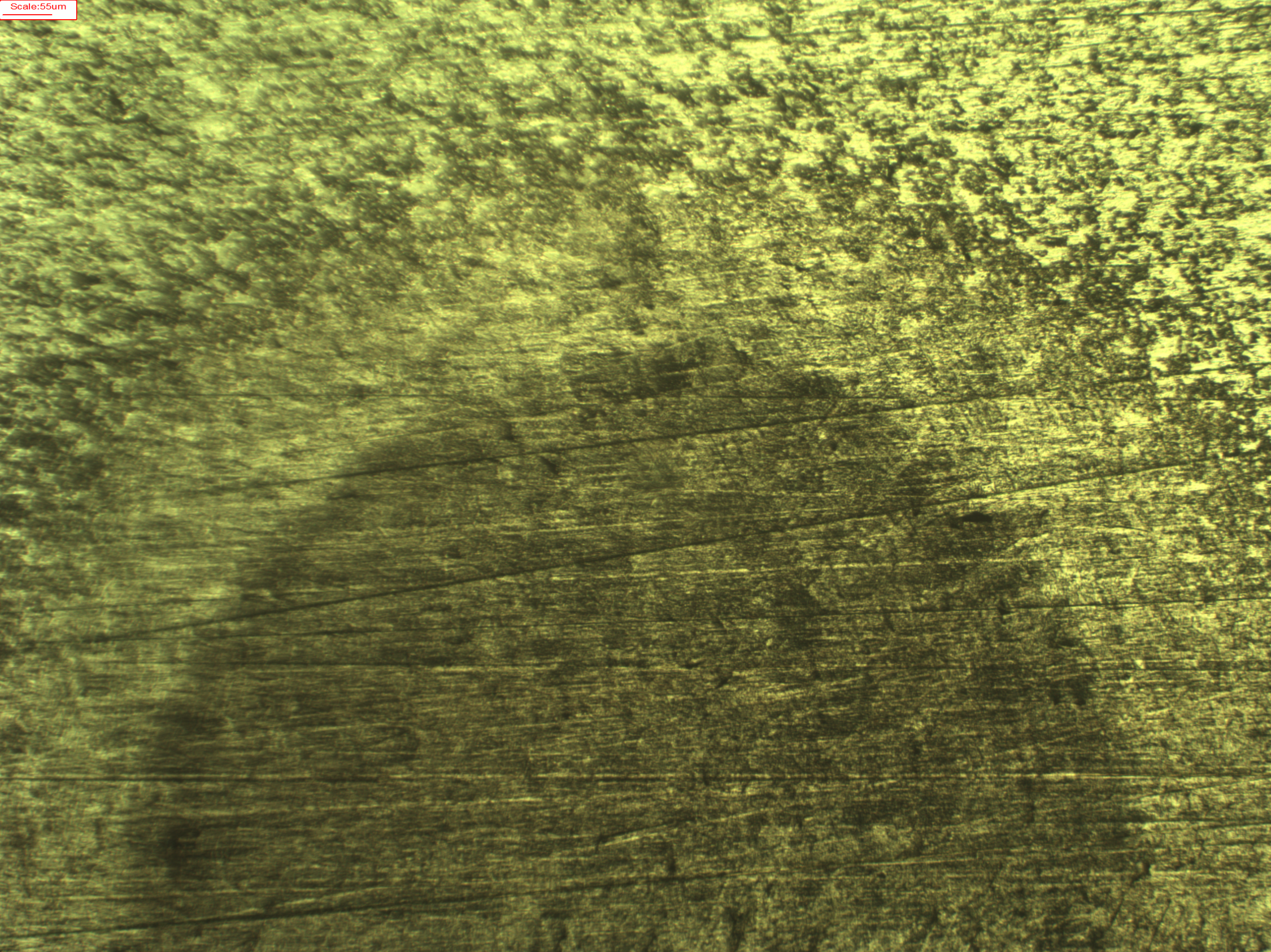

Supplement: S7 Fig — After quenching, the samples were polished and etched with 4% Nital solution. The microstructure of the quenched samples were obtained by the optical microscope named Oxion OX.2153-PLM EUROMEX, Holland. (TIF) [file pone.0314648.s007.tif]

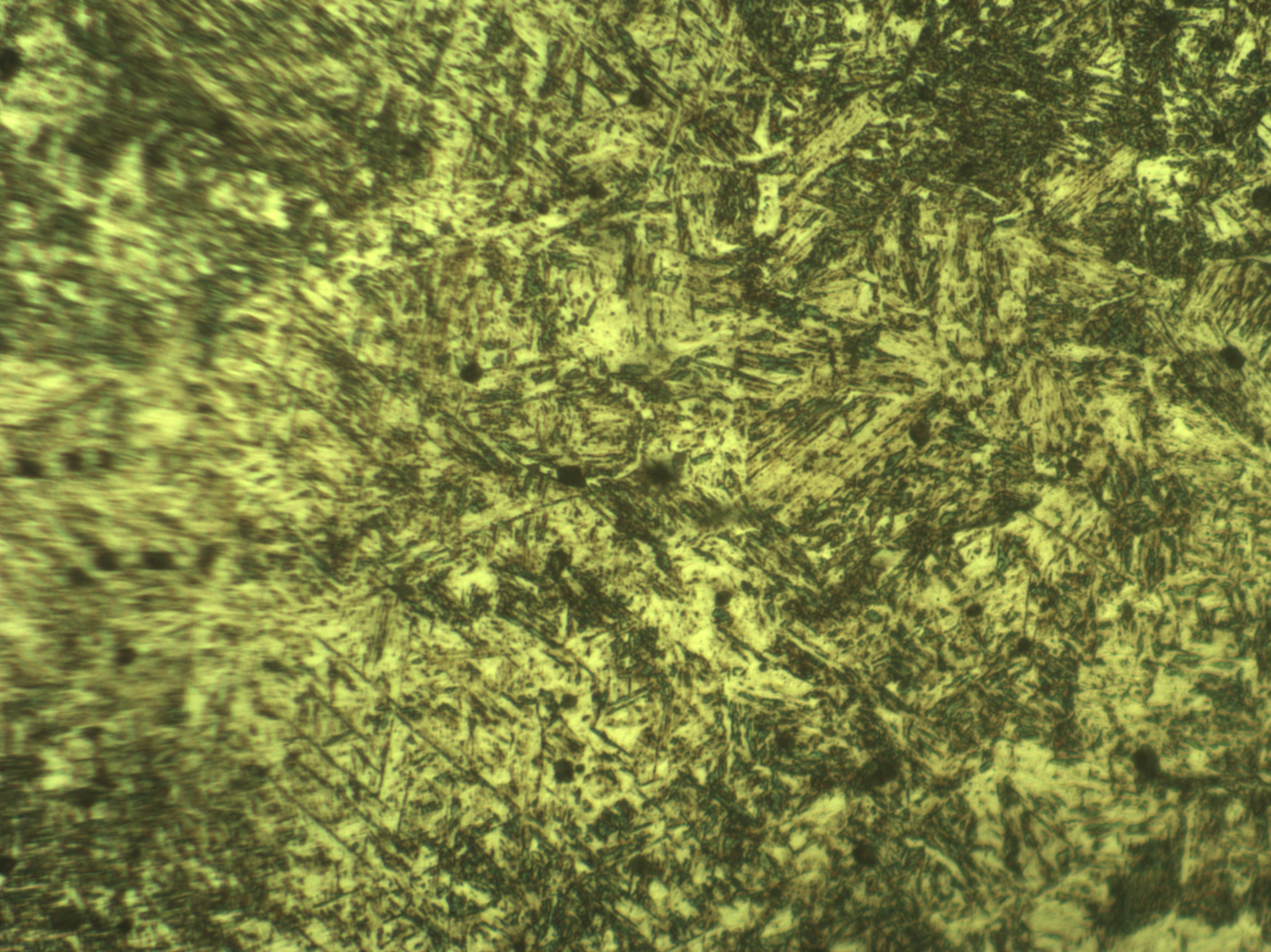

Supplement: S8 Fig — They were obtained by the optical microscope named Oxion OX.2153-PLM EUROMEX, Holland. (TIF) [file pone.0314648.s008.tif]

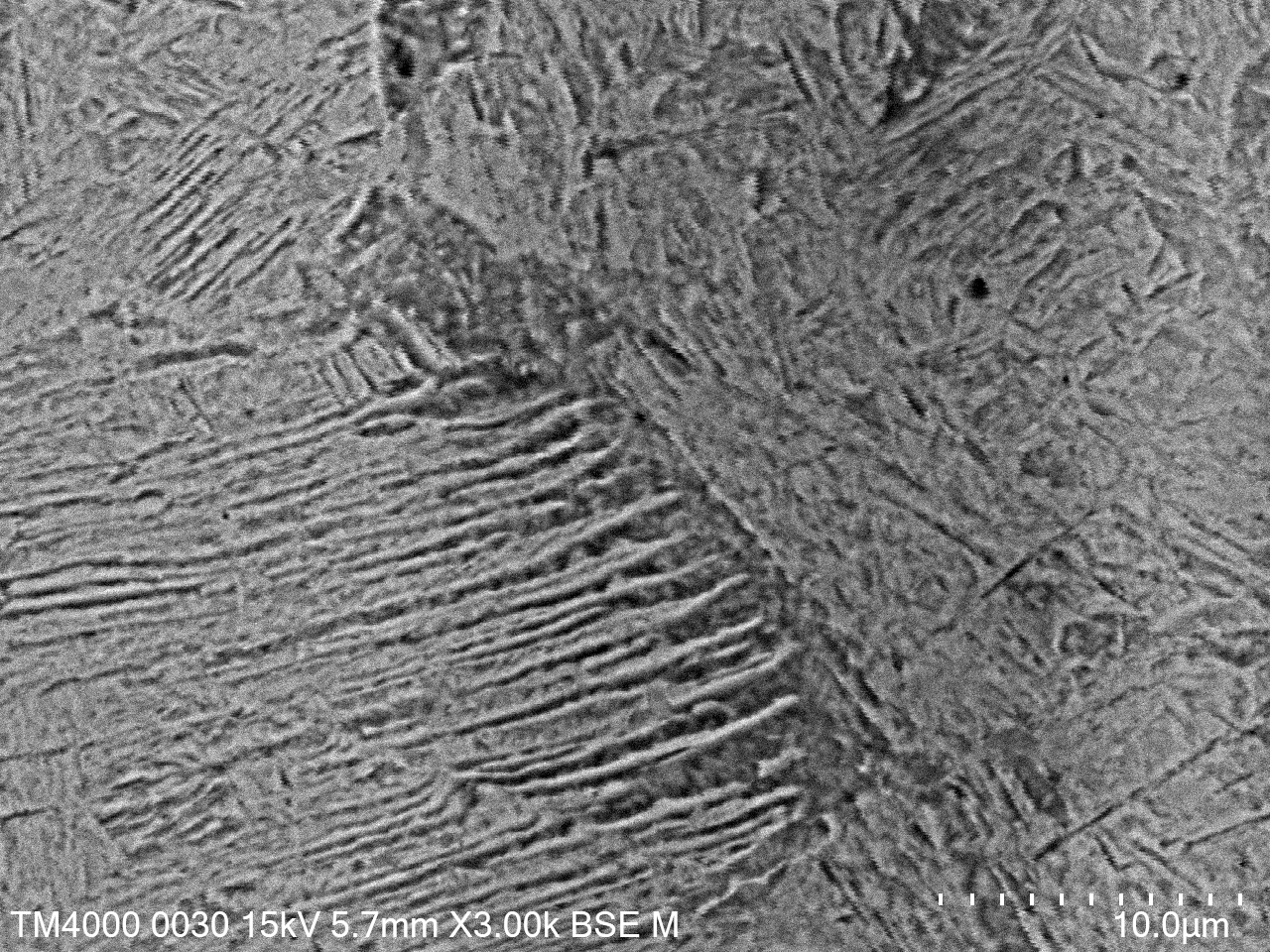

Supplement: S9 Fig — It was also observed via a scanning electron microscope (SEM) named JEOL 5410 LV, Japan. (TIF) [file pone.0314648.s009.tif]
